# Treatment with Raltegravir, a retroviral integrase inhibitor, in patients infected with HTLV-1

**DOI:** 10.1186/1742-4690-8-S1-A55

**Published:** 2011-06-06

**Authors:** Ana Treviño, Carmen de Mendoza, Patricia Parra, Jose M Eiros, Raul Ortiz de Lejarazu, Vincent Soriano

**Affiliations:** 1 Hospital Carlos III, Madrid, Spain; 2 Hospital Clínico Universitario, Valladolid, Spain

## Background

The HTLV-1 integrase enzyme shares important structural similarities with the HIV-1 integrase. Experimental studies have demonstrated that styrylquinolines and diketo acids can inhibit both HIV-1 and HTLV-1 integrases. Raltegravir is the only approved integrase inhibitor for the treatment of HIV infection. No data exist about its potential benefit in HTLV-1 carriers.

## Methods

A series of HTLV-1 individuals on regular follow-up at two Spanish clinics were invited to participate in a pilot trial in which raltegravir 40 mg bid was administered for 12 months. Proviral DNA was measured using a validated real-time PCR assay that targets the HTLV-1 pol region on PBMCs at baseline and periodically during follow-up. Demographics and clinical signs/symptoms were closely monitored in all patients.

## Results

A total of 5 HTLV-1 individuals entered the study. Their country of origin was Peru (2), Ecuador (1), Dominican Republic (1) and Colombia (1). Their median age was 52-years old. Three were women. All were infected with HTLV-1 subtype a subgroup a transcontinental. Route of contagion was heterosexual contact in 3, Transfusion in 1 and homosexual relationships in 1. The latest was coinfected with HIV-1. Two patients had TSP/HAM whereas the other 3 were asymptomatic.

Median proviral load was 1,658 HTLV-1 DNA copies per 10,000 PBMC in symptomatic patients compared to 758 copies in asymptomatic individuals. Following initiation of raltegravir therapy and up to 6 months, TSP patients experienced a transient decline in HTLV-1 proviral load (from 2,248 to 519 and from 1,033 to 861 copies per 10,000 PBMC, respectively). However, a return to baseline levels was seen in subsequent determinations, being proviral load at 12 months of 2,219 and 1,175, respectively. No improvement in clinical manifestations could be recognised. No significant changes in HTLV-1 proviral load were noticed in the 3 asymptomatic individuals with median proviral load values over time fluctuating around 755 copies /10,000 PBMCs.

## Conclusions

Treatment with raltegravir may produce a transient decline in HTLV-1 proviral load in TSP/HAM patients, which is not seen in asymptomatic carriers. However, there is no improvement in TSP/HAM clinical manifestations nor sustained virological benefit beyond six months of therapy.

